# Developing Novel Hydroxypropyl-β-Cyclodextrin-Based Nanosponges as Carriers for Anticancer Hydrophobic Agents: Overcoming Limitations of Host–Guest Complexes in a Comparative Evaluation

**DOI:** 10.3390/pharmaceutics14051059

**Published:** 2022-05-15

**Authors:** Shohreh Peimanfard, Ali Zarrabi, Francesco Trotta, Adrián Matencio, Claudio Cecone, Fabrizio Caldera

**Affiliations:** 1 Department of Biotechnology, Faculty of Biological Science and Technology, University of Isfahan, Isfahan 81746-73441, Iran; shohreh.peimanfard@gmail.com; 2 Department of Chemistry, University of Turin, Via Pietro Giuria 7, 10125 Torino, Italy; claudio.cecone@unito.it (C.C.); fabrizio.caldera@unito.it (F.C.); 3 Department of Biomedical Engineering, Faculty of Engineering & Natural Sciences, Istinye University, Sariyer, Istanbul 34396, Turkey

**Keywords:** 2-hydroxypropyl-β-cyclodextrin, nanosponge, host–guest complex, naringenin, drug delivery systems, cytotoxicity

## Abstract

This study aimed to design and fabricate novel hydroxypropyl-β-cyclodextrin-based hypercrosslinked polymers, called nanosponges, as carriers for anticancer hydrophobic agents and compare them with host–guest complexes of hydroxypropyl-β-cyclodextrin, a remarkable solubilizer, to investigate their application in improving the pharmaceutical properties of the flavonoid naringenin, a model hydrophobic nutraceutical with versatile anticancer effects. For this purpose, three new nanosponges, crosslinked with pyromellitic dianhydride, citric acid, and carbonyldiimidazole, were fabricated. The carbonate nanosponge synthesized by carbonyldiimidazole presented the highest naringenin loading capacity (≈19.42%) and exerted significantly higher antiproliferative effects against MCF-7 cancer cells compared to free naringenin. Additionally, this carbonate nanosponge formed a stable nanosuspension, providing several advantages over the naringenin/hydroxypropyl-β-cyclodextrin host–guest complex, including an increase of about 3.62-fold in the loading capacity percentage, sustained released pattern (versus the burst pattern of host–guest complex), and up to an 8.3-fold increase in antiproliferative effects against MCF-7 cancer cells. Both naringenin-loaded carriers were less toxic to L929 murine fibroblast normal cells than MCF-7 cancer cells. These findings suggest that hydroxypropyl-β-cyclodextrin-based carbonate nanosponges could be a good candidate as a drug delivery system with potential applications in cancer treatment.

## 1. Introduction

Scientific evidence demonstrates that considerable numbers of current chemotherapeutic agents are directly derived from plants or semi-synthesized using plant materials. Therefore, the use of botanicals in chemotherapy is inevitable [1]. In recent years, over 4000 different types of flavonoids identified in the human diet have attracted interest for the development of health-promoting and therapeutic plant-derived compounds [2]. Many studies have shown the antioxidant and anticancer effects of flavonoids, indicating their potential to be developed as promising nutraceuticals and therapeutic agents with minimal side effects [3].

Naringenin (NG) (4′, 5, 7-trihydroxyflavanone), is a flavonoid that is found in citrus fruits (grapefruits and oranges in particular) and tomatoes. NG, as a bioactive plant compound and a frequent component in the human diet, has attracted attention for cancer prevention and therapy. In various studies, carcinogen inactivation, induction of apoptosis, cell cycle arrest, inhibition of sustained angiogenesis, and antiproliferative effects against different cancer cells have been reported [3,4,5]. Despite its beneficial properties, NG suffers from low aqueous solubility (about 36 µM) and minimal bioavailability (5.8%) [6], which could restrict its usage.

From a pharmaceutical standpoint, one of the useful functional choices for improving the aqueous solubility and bioavailability of hydrophobic compounds is the use of cyclodextrins (CDs). CDs are a family of cyclic oligosaccharides composed of (α-1,4)-linked D-glucopyranose units that form a truncated cone or torus shape due to the chair conformation of the glucopyranose molecules. Owing to a hydrophobic central cavity and hydrophilic outer surface, CDs can interact as a host with many hydrophobic guest compounds, resulting in improvements in their aqueous solubility [7]. α-Cyclodextrin (αCD), β-cyclodextrin (βCD), and γ-cyclodextrin (γCD) are the three parents CDs on the FDA’s generally recognized as safe (GRAS) list of food additives [8]. Up to 2007, 35 different drugs were marketed as solid or solution-based CD complex formulations; subsequently, the number of CD-containing pharmaceutical products increased to more than 84 in 2019 [9]. The currency of CDs as useful solubilizing excipients has been evolving over the past decades owing to their ever-increasing advantageous properties and applications [10]. 2-Hydroxypropyl-β-cyclodextrin (HPβCD), as a hydrophilic derivative of βCD, is much more water-soluble and more toxicologically benign than the parent βCD [11]. It has undergone extensive safety studies and is marketed in several drug formulations e.g., itraconazole (Sporanox^®^, Janssen, Europe, and the USA) with oral dosing of up to 8 g HPβCD/day and intravenous dosing of up to 16 g HPβCD/day [7]. Despite the critical properties of HPβCD in improving the solubility and bioavailability of the hydrophobic components, as well as its nontoxicity, the the main obstacles in pharmaceutical applications are the rather low complexation efficiency and loading capacity, similar to other members of the CD family. Usually, solid drug/cyclodextrin complexes comprise less than 5 to 10% of the drug [12,13]. Therefore, a large number of CDs are needed to complex a rather small mass of a drug that can negatively affect formulation bulk, product cost, and even toxicological considerations [13]. The crosslinking of CDs to form porous three-dimensional hypercrosslinked polymers called nanosponges presents a functional approach to CD-based drug delivery systems with higher loading capacities.

CDs, as polyfunctional monomers with reactive hydroxyl groups, can be crosslinked with a variety of crosslinkers, including pyromellitic dianhydride (PMDA) and citric acid to form ester nanosponges, and 1,1′-carbonyldiimidazole (CDI) to form carbonate nanosponges [14]. In addition to the internal cavities of CDs, nanosponges possess many interstitial cavities by crosslinking several CD molecules, providing more to accommodate a higher quantity and wider series of cargos in comparison with the respective native CDs. Moreover, surrounding the nanosponges cavities by the polymer network hampers the burst release of the entrapped cargos and can promote the slower release [14]. Nanosponges prepared by various crosslinkers have been demonstrated to ameliorate some pharmaceutical properties (e.g., loading capacity, drug release profile, stability, and cytotoxicity) of different drugs such as resveratrol [15], tamoxifen [16], paclitaxel [17,18], camptothecin [19], quercetin [20], and curcumin [21]. In a recent study, Argenziano et al. demonstrated that doxorubicin-loaded nanosponges could inhibit the proliferation of breast cancer cells with greater efficiency than free doxorubicin. Furthermore, 60% inhibition of breast cancer growth in BALB-neuT mice was obtained by a five-fold lower dose of doxorubicin-loaded nanosponges than the doxorubicin therapeutic dose. These results revealed that nanosponges could be considered an ideal drug delivery system for breast cancer treatment [22].

CDI and PMDA have been widely employed as a crosslinker for the synthesis of β-cyclodextrin nanosponges to be applied as a drug delivery system, especially for cancer treatment. Krabicová et al. comprehensively overviewed many in vitro and in vivo studies on β-cyclodextrin nanosponges in the last decades and concluded that β-cyclodextrin nanosponges crosslinked with CDI or PMDA could improve the desired pharmaceutical properties in wide range of guest molecules while no evidence of toxicity has been reported for the blank nanosponges [23]. Moreover, in recent years, citric acid has attracted the attention as a crosslinker for the synthesis of β-cyclodextrin nanosponges because of the associated eco-friendly synthesis process in which water is the only solvent used [24]. Besides, in a previous work, citric acid was successfully used as a crosslinker to fabricate β-cyclodextrin nanosponges for application as a safe nontoxic excipient in tablet design [25]. All these aforementioned benefits suggest the value of using these crosslinkers for designing novel nanosponges.

This study aimed to exploit HPβCD under two conditions: first, as a solubilizer in a host–guest complex to investigate if the remarkable solubilizing potential of HPβCD could result in improved anticancer effects of NG; and second, as a building block for the fabrication of new nanosponges to investigate the application of HPβCD-based nanosponges as drug delivery systems for improving the release profile and the antiproliferative effects of NG compared to NG/HPβCD host–guest complexes. For this purpose, three types of HPβCD-based nanosponges were fabricated, and the nanosponge with the highest NG loading capacity was compared with the NG/HPβCD host–guest complex for loading capacity, particle size, morphology, stability, solubility, release profile, and increased antiproliferative effects compared with free NG. Their structures were characterized through TGA, DLS, ATR-FTIR, ^1^H NMR, ^13^C CPMAS solid-state NMR, and SEM.

## 2. Materials and Methods

### 2.1. Materials

2-Hydroxypropyl-β-cyclodextrin (average molecular weight = 1396 g mol^−1^) naringenin (≥95% purity), pyromellitic dianhydride (PMDA), dimethyl sulfoxide (DMSO), triethylamine (TEA), 1,1′-carbonyldiimidazole (CDI), dimethylformamide (DMF), citric acid, and sodium hypophosphite monohydrate (SHP), ethanol absolute, and ethyl acetate ≥99.5% were purchased from Sigma-Aldrich (Darmstadt, Germany). MCF-7 and L-929 cell lines were obtained from the Pasteur Institute of Iran, Tehran, Iran. The cell culture reagents were received from Gibco/Invitrogen (Life Technologies, Paisley, UK). All reagents were of analytical grade. Milli-Q^®^ water was obtained using a Millipore Direct-Q^®^ 5 production system and was used throughout the study.

### 2.2. Phase Solubility Study

The phase solubility analysis was performed according to the method of Higuchi and Connors [26,27]. Briefly, an excess amount of NG was added into several vials containing different concentrations of HPβCD aqueous solution (from 0 to 322 mM). The vials were agitated by a magnetic stirrer in the dark at room temperature for 72 h. Finally, the suspensions were centrifuged at 14,000 rpm for 30 min at 4 °C and the supernatants were analyzed by UV spectrophotometer (PerkinElmer Lambda 25, Waltham, MA, USA) at 288 nm to determine total NG concentration. All experiments were performed in triplicate and the data were presented as the mean value ± the standard deviation (SD).

The phase solubility profile was then constructed by plotting the graph of NG concentration against HPβCD concentration. Assuming NG:HPβCD 1:1 complex formation (Equation (1)), the apparent stability constant (K_1:1_) and the complexation efficiency (CE) were calculated based on the slope of the linear solubility–phase profile through Equations (2) and (3), respectively [26,27]. Then, increase in formulation bulk was calculated based on CE and molecular weights of NG and HPβCD by Equation (4) [28].

NG + HPβCD ⇌ NG.HPβCD complex(1)

K_1:1_ = [NG.HPβCD complex]/[NG] × [HPβCD] = slope/NG_0_ × (1 − slope) (2)

CE = [NG.HPβCD complex]/[HPβCD] = NG_0_ × K_1:1_ = slope/(1 − slope) (3)

Increase in formulation bulk = (MW_HPβCD_/MW_NG_) × (1 + (1/CE)) (4)

where NG_0_ is the intrinsic solubility of NG in distilled water, [NG.HPβCD complex] is the concentration of dissolved complex, and [HPβCD] is the concentration of free HPβCD in the aqueous complexation medium, and MW_HPβCD_ and MW_NG_ are molecular weights of HPβCD and NG, respectively.

### 2.3. Preparation of the NG/HPβCD Complex

**Figure 1:**
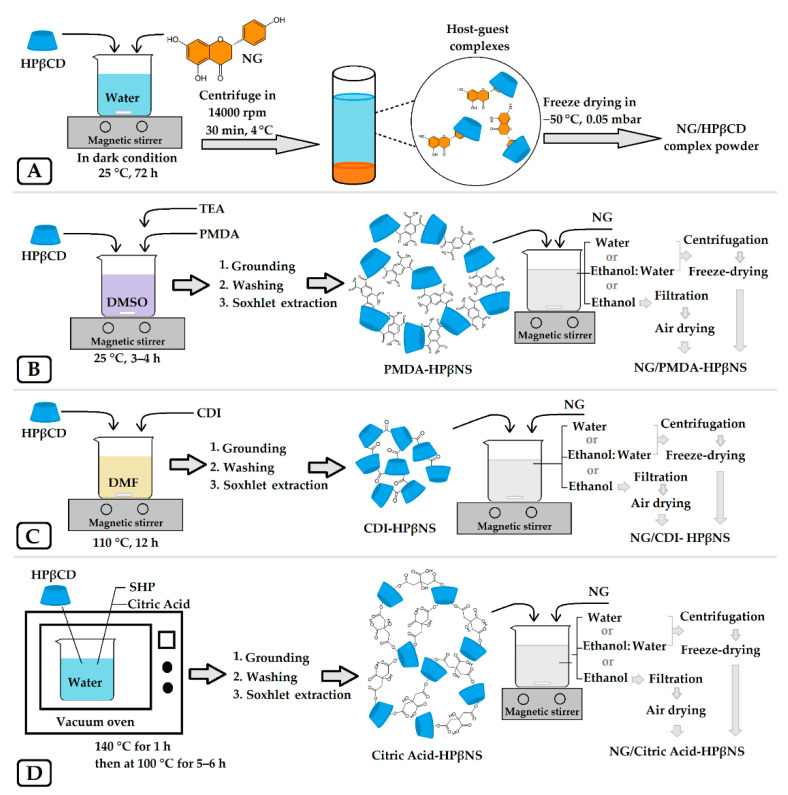
The schematic diagram of NG/HPβCD complex preparation (**A**), synthesis of nanosponges, and NG loading with crosslinkers of PMDA (**B**), CDI (**C**), and citric acid (**D**).

According to the phase solubility results, the HPβCD concentration leading to the maximum NG aqueous solubility was chosen for NG/HPβCD complex preparation. The schematic of NG/HPβCD complex preparation is illustrated in Figure 1A.

For this purpose, excess NG (28 mg/mL) was added to an aqueous solution of HPβCD (449.51 mg/mL) under continuous magnetic stirring in the dark at room temperature for 72 h. Finally, the suspensions were centrifuged at 14,000 rpm for 30 min at 4 °C. The obtained supernatant was lyophilized using a lyophilizer (Telstar^®^, Tokyo, Japan) at −50 °C and operating pressure of 0.05 mbar. The lyophilized NG/HPβCD complex powder was stored in sealed tubes at 4 °C for use in the next experiments.

### 2.4. Fabrication of HPβCD-Based Nanosponges and NG Loading

Three types of HPβCD-based nanosponges (HPβNS) were synthesized using three different crosslinkers based on the previously reported methods and their schematic diagrams are illustrated in Figure 1B–D [24,29,30]. To the best of our knowledge, this is the first time that nanosponges have been fabricated with HPβCD as the only monomer and with PMDA, citric acid, and CDI crosslinkers. The accurate amounts and conditions needed for each type of nanosponge are revealed in Table 1. For the synthesis procedure, the selected values of anhydrous HPβCD were dissolved in an accurate amount of the suitable solvent. Then, the appropriate crosslinker and catalyst were added to the solution and allowed to react with it in their suitable condition (Table 1). The obtained monolithic mass of the nanosponge was crushed and ground in a mortar. Then, they were washed with deionized water, followed by ethanol or acetone, to remove unreacted components. The purified nanosponges were obtained by Soxhlet extraction with ethanol or acetone for 72 h, air-dried, and stored in a vacuum desiccator at room temperature until further use.

In next step, three different types of nanosponges were loaded with NG via three different solvents as presented in the scheme in Figure 1B–D. Two types of NG-loaded HPβNS were prepared by the following freeze-drying method [31]: NG (10 mg/mL) was added to a suspension of each HPβNS (5 mg/mL) in two different solvents, i.e., pure water and water/ethanol (50% *v*/*v*)*,* under magnetic stirring for 48 h in the darkness at room temperature (1:2 *w*/*w* HPβNS:NG). The supernatant was separated through centrifugation at 4500 rpm for 30 min and was lyophilized using Telstar lyophilizer at −50 °C and operating pressure of 0.05 mbar for use in subsequent experiments.

The third type of NG-loaded HPβNS was prepared by soaking an accurate amount of each HPβNS (5 mg/mL) in a solution of NG in ethanol (10 mg/mL) under continuous magnetic stirring for 48 h in the dark (1:2 *w*/*w* HPβNS:NG). Since all HPβNS types are insoluble in ethanol, NG-loaded HPβNS were recovered through filtration and left to dry for 72 h at 25 °C. All nine NG-loaded HPβNS powders obtained were stored in sealed tubes at 4 °C for further analysis.

### 2.5. Determination of NG Loading Capacity in NG/HPβCD Complex and NG-Loaded HPβNS

Twenty milligrams of NG/HPβCD complex and every NG-loaded HPβNS was dispersed in 10 mL of ethyl acetate, sonicated for 1 h, and then stirred overnight to release the entrapped NG. The concentration of released NG was analyzed by UV–Vis spectrophotometer (PerkinElmer Lambda 25, Waltham, MA, USA) at 288 nm. The experiments were performed in triplicate and the data were presented as the mean ± SD. The loading capacity of NG was calculated by the following formula.

NG loading capacity % = Mass of entrapped NG/Initial mass of HPβCD or HPβNS × 100 (5)

### 2.6. Preparation of the Physical Mixture

According to the loading capacity results, the physical mixture was prepared in almost the same weight ratio of NG to HPβCD (i.e., 1:20) and NG to HPβNS (i.e., 1:5) by carefully mixing in a mortar to make a homogeneous mixture for use in the characterization tests.

### 2.7. Physicochemical Characterization

#### 2.7.1. Thermogravimetric Analysis

Thermogravimetric analysis (TGA) was performed on a TA Instruments Q500 TGA (New Castle, DE, USA), over a temperature range from 0 °C to 700 °C, under nitrogen flow, with a heating rate of 10 °C/min.

#### 2.7.2. Determination of Particle Size, Polydispersity Index, and Zeta Potential

NG-loaded carriers were diluted suitably by Milli-Q water (1 mg/mL) and analyzed for particle size, polydispersity index (PDI), and zeta potential by dynamic light scattering using a Brookhaven Instrument (90Plus Particle Size Analyzer, NY, USA) at the fixed scattering angle of 90° at 25 °C. The analysis was performed instantly after sample preparation (0 h) and again after 7 days of storage at room temperature without agitation (7d). All measurements were conducted in triplicate and the data were reported as mean ± SD.

#### 2.7.3. Fourier Transform Infrared Spectroscopy

ATR-FTIR spectra of pure NG, blank carriers, their physical mixtures, and NG-loaded carriers were obtained by a Perkin Elmer Spectrum 100 FT-IR Spectrometer (Waltham, MA, USA) equipped with a Universal ATR Sampling Accessory used for attenuated total reflection (ATR-FTIR) spectroscopy in the region of 4000–650 cm^−1^ at room temperature with a resolution of 4 cm^−1^ and 8 scans/spectrum.

#### 2.7.4. ^1^H NMR and ^13^C Cross-Polarization Magic Angle Spinning Solid-State NMR

^1^H NMR spectra for NG, HPβCD, and NG/HPβCD were recorded by a Bruker AVANCE 400 spectrometer (Bruker BioSpin GmbH, Karlsruhe, Germany) with DMSO as solvent. ^13^C Cross-Polarization Magic Angle Spinning (CPMAS) solid-state NMR spectra for NG, nanosponges, and NG-loaded nanosponges were acquired with a Jeol ECZR 600 instrument operating at 600.17 and 150.91 MHz for ^1^H and ^13^C nuclei, respectively. Powder samples were packed into cylindrical zirconia rotors with a 3.2 mm OD and a 60 μL volume. A certain amount of sample was collected from each batch and used without further preparations to fill the rotor. ^13^C CPMAS spectra were acquired at a spinning rate of 20 kHz using a ramp cross-polarization pulse sequence with a contact time of 3.5 ms, a 90° ^1^H pulse of 2.1 μs, (optimized) recycle delays between 1.7 and 95 s, and several scans, between 50 and 2250 depending on the sample. For every spectrum, a two-pulse phase modulation (TPPM) decoupling scheme was used with a radiofrequency field of 108.5 kHz. The chemical shift scale was calibrated through the methylenic signal of external standard glycine (at 43.7 ppm).

#### 2.7.5. Particle Morphology by Scanning Electron Microscopy

The morphological features of free NG, free carriers, drug-loaded carriers, and their physical mixtures were examined by scanning electron microscopy (Leica Stereoscan 410 Oxford Instrument, Abingdon, UK), at a 10 kV accelerating voltage. Before performing SEM, the samples were gold-coated using a Baltec SCD 050 sputter coater (Pfäffikon, Switzerland) for 40 s, under vacuum, at 60 mA.

### 2.8. In Vitro Release Study

In vitro release study was performed based on an ultrafiltration technique previously recommended by Wallace et al. as the optimal method of choice for the release study of nanomedicine carriers [32]. Briefly, 20 mg of NG-loaded carriers were dispersed into 20 mL of phosphate-buffered saline under magnet stirring in 2 different conditions: 37 °C in pH 7.4 and 39 °C in pH 6.6. At predetermined interval times, a 5 mL aliquot was withdrawn and replaced with the same amount of fresh buffer solution to keep the sink condition. The aliquots were filtered through the ultrafiltration process using an Amicon 8050 50 mL stirred ultrafiltration cell, fitted with a 44.5 mm diameter regenerated cellulose YM1 ultrafiltration membrane (MWCO 1 kDa, Millipore Corp., Bedford, MA, USA). The pressure was exerted by nitrogen (<20 psi) while the solution was stirring using the inbuilt magnetic stirrer. The ultrafiltrate was collected for determination of NG concentration by UV-Vis spectrophotometer (PerkinElmer Lambda 25, Waltham, MA, USA) at 288 nm. All experiments were performed in triplicate and the data were presented as mean ± SD.

### 2.9. Cytotoxicity Studies

The inhibition percentage of cell viability by free NG and the carriers (with/without drug components) were evaluated using 3-(4,5-dimethylthiazol-2-yl)-2,5-diphenyltetrazolium bromide (MTT) assay in the MCF-7 breast cancer cell line and the L929 murine fibroblast cell line as cancerous and normal cells, respectively.

Cells (10,000 cells/well) were cultured into 96-well plates containing Dulbecco’s modified Eagle medium (DMEM) and incubated for 24 h at 37 °C in a 5% CO_2_ atmosphere. The cells were then treated with different concentrations of NG as the positive control (dissolved in DMSO and then dispersed in DMEM at 0.1% (*v*/*v*) final concentration), blank nanocarrier, and drug-loaded ones for 96 h (8–64 µg/mL). Subsequently, the medium was removed and the cells were washed twice with PBS. Then, each well was supplemented by 100 μL of fresh medium with 10 μL MTT solution (5 mg/mL) and incubated for 4 h at 37 °C. Then, the medium in each well was replaced by 100 μL DMSO and, after incubating for 1 h, the absorbance of the wells was recorded at 493 nm using an ELISA reader (Bio-Rad, Hercules, CA, USA). The experiment was performed in triplicate for each well and the data were presented as the mean ± SD [33].

### 2.10. Statistical Analysis

Data were expressed as mean ± standard deviation (SD) for each group. Significant difference among the experimental groups was demonstrated by one-way ANOVA followed by Tukey multiple comparisons as the post hoc test (according to the homogeneity of variances) using SPSS software (Version 16.0. SPSS Inc. Chicago, IL, USA). A *p*-value < 0.05 was considered to represent statistical significance and indicated by an asterisk (* *p* < 0.05).

## 3. Results and Discussion

### 3.1. Phase Solubility Studies

**Figure 2:**
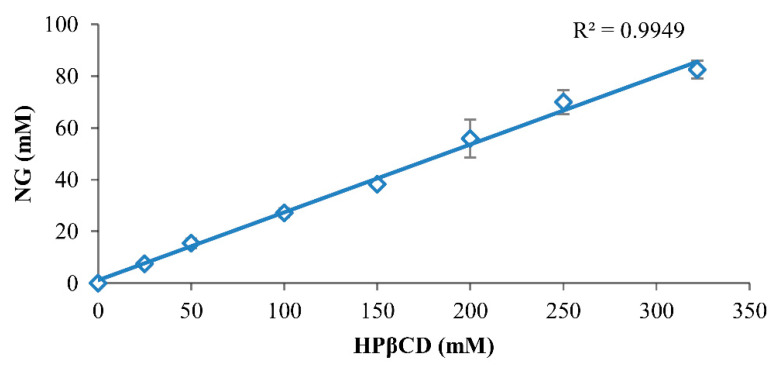
Phase solubility profile of NG in pure aqueous medium at room temperature (mean ± standard deviation; *n* = 3).

The phase solubility profile by the Higuchi–Connors method demonstrated the formation of NG/HPβCD complexes of A_L_-type and indicated that HPβCD solubilizes NG as a linear function of its concentration (Figure 2). This means that when an NG/HPβCD solution is administered (e.g., by oral or intravenous dosing), both NG and HPβCD concentrations are decreased in a linear manner and precipitation is not likely to occur. However, it is worth mentioning that organic solvents solubilize solutes as a log function of their concentration; therefore, by exposing them to aqueous medium, the solubilizing strength of the formulation is quickly lost and precipitation occurs. This could demonstrate the superiority of solubilizing NG by CDs in comparison with using organic solvents in pharmaceutical applications [7,27]. As can be seen in the Higuchi–Connors profile, the complex formation of HPβCD with NG has a tremendous effect on the water solubility of NG, and a saturated concentration of HPβCD in water (almost 322 mM) could increase the NG aqueous solubility by about 2375 fold.

The observed stability constant (K_1:1_) in the Higuchi–Connors method could estimate the strength of the guest–CD interaction representing several guest solubilizing mechanisms that exist together in non-ideal aqueous cyclodextrin solutions [27]. The calculated K_1:1_ based on the Higuchi–Connors method (Equation (2)), indicated a high stability constant, up to 11 × 10^3^ M^−1^, which showed the high affinity of NG for HPβCD.

During formulation work, it is often more suitable to compare the CE than K_1:1_ values because CE is less sensitive to errors associated with the determination of intrinsic drug solubility [7]. Therefore, the complexation efficiency (CE ≈ 0.35) (Equation (3)) is a more accurate parameter to show the solubilizing effect of HPβCD on NG, independent of its intrinsic solubility in water (NG_0_). From Equation (3), the molar ratio of NG/HPβCD complex to free HPβCD is estimated to be 1:3. In other words, only about one out of four HPβCD molecules may form a complex with NG and the other three HPβCD molecules are free. This CE leads to an almost 19.79-fold increase in dosage bulk based on the Equation (4). The feasibility of formulating a given pharmaceutical compound as a cyclodextrin complex depends on the CE and dosage bulk. Potent compounds with high CE and reasonable dosage bulk are suitable for cyclodextrin formulations [13,34]. High CE value and appropriate increases in dosage bulk suggested that the NG/HPβCD complex has good potential for use as a solid dosage form.

### 3.2. Determination of NG Loading Capacity in NG/HPβCD Complex and NG-Loaded HPβNS

NG loading capacity was determined to compare the amount of NG loaded per unit weight of the nanocarriers. The NG/HPβCD complex had a loading capacity of 5.36 ± 0.32% NG, which is common among cyclodextrin–guest complexes. Since the loading capacity has been defined based on the mass ratio of the guest to the host (i.e., CD) and the molecular weight of guest is usually much less than that of the CD, the loading capacity is mostly limited to around 5–10%, even in 1:1 host–guest complexes [35].

The results of NG-loaded nanosponges demonstrated that water and water/ethanol (50:50 *v*/*v*) are not appropriate solvents for NG loading in any type of synthesized HPβNS (Table 2), which might be due to incomplete solubilizing of NG in these solvents. The percentage of NG loading capacity in PMDA-HPβNS was less than 3% in water and water/ethanol and was not detectable in pure ethanol because the NG-loaded PMDA-HPβNS became aggregated and sticky after exposure to the solvent. However, pure ethanol could be a good solvent for NG loading in CDI-HPβNS and citric acid-HPβNS since it could increase the NG loading capacity significantly more than two other solvents (*p* < 0.0001). It could be because ethanol solubilizes NG completely and disperses the molecules, allowing entry to the cavities of nanosponges. Moreover, the NG loading capacity of CDI-HPβNS was about four-fold higher compared to citric acid-HPβNS (*p* < 0.0001). Therefore, among all synthesized HPβNS types, NG-loaded-CDI-HPβNS (NG/CDI-HPβNS) was selected for the subsequent experiments.

### 3.3. Physicochemical Characterization 

#### 3.3.1. Thermogravimetric Analysis

**Figure 3:**
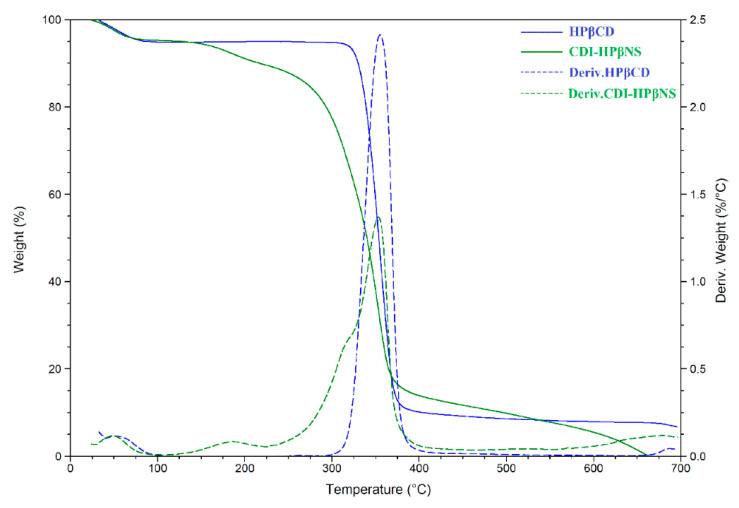
Thermogravimetric analyses of HPβCD (TGA in blue solid line and DTG in blue dashed line), CDI-HPβNS (TGA in green solid line and DTG in green dashed line).

TGA and its corresponding derivative (DTG) curves were recorded to analyze and differentiate the CDI-HPβNS from HPβCD (as its monomer) through changes in their thermal degradation paths (Figure 3). HPβCD exhibited a two-step mass loss as expected [36]: the first step due to vaporization of adsorbed environmental water at temperatures up to 100 °C, which is a typical phenomenon for hygroscopic materials; the second step owing to decomposition of HPβCD starting at around 300 °C with the maximum mass-loss rate at about 350 °C.

Three distinct mass-loss phenomena could be observed in the TGA curve of CDI-HPβNS (Figure 3, green solid line) with the maximum mass-loss rate at about 50 °C, 190 °C, and 350 °C, as evidenced by the DTG curve (Figure 3, green dashed line). The initial mass loss could be attributed to evaporation of adsorbed water. similar to HPβCD and hygroscopic CD-based materials. The second mass loss, with the maximum mass-loss rate at 190 °C, might be attributed to the condensation reaction by reactive pendants that can occur at this temperature and resulted in condensing free reactive carbonyl groups to carbonate bridges. This is in agreement with a previous report for crosslinked βCD polymers, which indicated the condensation of free carbonyl groups to ester bridges resulting in a mass loss at around 180 °C [24]. The third mass-loss step started at around 225 °C and reached its maximum rate at 350 °C, which aligns with the previous studies on nanosponges [24,37]. This could be related to the thermal degradation of carbonate bridges in the CDI-HPβNS structure, which then result in carbon residue by the pyrolysis of HPβCD components of the system.

#### 3.3.2. Determination of Particle Size, Polydispersity Index, and Zeta Potential

The average particle size, PDI, and zeta potential of NG/HPβCD and NG/CDI-HPβNS in Milli-Q water at the time of preparation (0 h) and after 7 days of storage at room temperature are shown in Table 3. It has been well documented that DLS is an effective method for detecting HPβCDs aggregates and estimating their hydrodynamic diameter [38]. The DLS analysis indicated that the average hydrodynamic diameter of the NG/HPβCD complex aggregates was in the range of 42.33 ± 11.16 nm (at time 0 h) while after 7 days, the size of aggregates showed an almost two-fold increase (84.03 ± 13.46 nm). This result could be explained by several factors. First of all, cyclodextrins are known to self-assemble in an aqueous solution to form aggregates ranging from 20 nm to a few micrometers [38,39]. Moreover, Sá Couto et al. earlier demonstrated that after one week storage at 25 °C, free HPβCD aqueous solution (10% *w*/*v*) displayed a four-fold increase in the size of HPβCD aggregates. Moreover, their study clearly showed that the self-assembly of HPβCDs is a reversible process that could be affected by the concentration, filtration, and storage time [38]. Further, the almost neutral zeta potential of the NG/HPβCD complex (1.91 ± 0.29 at 0 h to 5.01 ± 0.37 at 7d) may more clearly indicate why the NG/HPβCD complex self-assembled and aggregated more during a week of storage without agitation. It should be mentioned that the detection and size estimation of these water-soluble aggregates are important since it demonstrated that the hydrated drug/cyclodextrin complexes are not separate and could interact with each other to form aggregates. In this way, they are able to solubilize lipophilic water-insoluble drugs through non-inclusion complexation [40].

In contrast, the zeta potential data for the NG/CDI-HPβNS at time zero (−21.63 ± 2.14) and after 7 days of storage (−26.03 ± 3.42) were in the desired range to form a stable nanosuspension and did not show any significant change during a week. The particle size of NG/CDI-HPβNS slightly increased after a week of storage from 428.33 ± 1.99 nm to 453.13 ± 20.26 nm. In an earlier study on CDI-based nanosponges, Dhakar et al. demonstrated that both blank nanosponges and kynurenic acid-loaded nanosponges did not undergo any significant changes in particle size and zeta potential during a week of storage at 4 °C [33]. The particle diameter distribution was also observed in the desired range for both NG-loaded carriers. This indicated that the PDI value of about 0.29 for NG/HPβCD arose from two factors: first, the self-assembly of HPβCD molecules by weak intermolecular forces in a reversible process, as demonstrated earlier, can lead to a moderate polydispersity of NG/HPβCD particles; second, the inclusion of NG in HPβCD cavities is also a reversible equilibrium (Equation (1)) governed mostly by van der Waals forces that can cause slight dispersity in particle sizes and increase the PDI [7]. The latter can also affect the PDI value of NG/CDI-HPβNS particles (0.26–0.27) as previously reported [33].

These results suggested that CDI-based nanosponges may be less prone to aggregation during storage and that they possess good potential for use as stable nanosuspensions.

#### 3.3.3. Fourier Transform Infrared Spectroscopy

**Figure 4:**
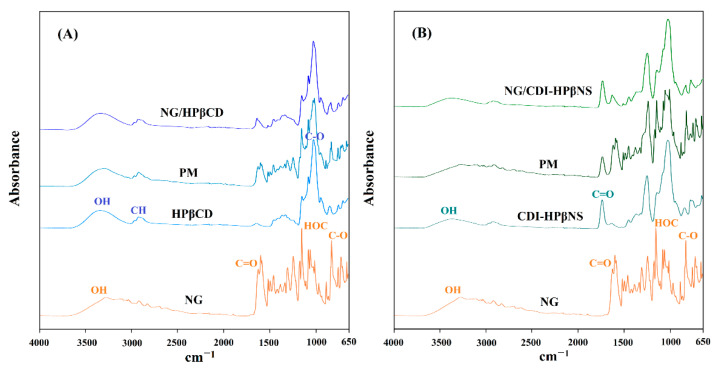
ATR-FTIR spectra of (**A**) NG, HPβCD, NG + HPβCD physical mixture (PM), and NG/HPβCD complex and (**B**) NG, CDI-HPβNS, NG + CDI-HPβNS physical mixture (PM), and NG/CDI-HPβNS.

The ATR-FTIR spectrum of NG (Figure 4A) indicated the characteristic vibrations at wavenumbers of 3280, 1626, 1154, 1081, and 830 cm^−1^, which could be assigned to O-H stretching, C=O stretching, HOC bending (ring A), C-O stretching (ring B), and C-O stretching (ring A), respectively, and were in agreement with a previously reported NG spectrum [41].

As shown in other studies, HPβCD (Figure 4A) demonstrated characteristic vibrations of saccharides at about 3343 cm^−1^ (O-H stretching), 2924 cm^−1^ (C-H stretching), 1645 cm^−1^ (O-H bending), and 1025 cm^−1^ (C-O stretching) [42]. Furthermore, the characteristic peak of the α-type glycosidic bond was observed at 851 cm^−1^, indicating the presence of glucopyranose units by the α-1,4-glycosidic bond. The peaks at about 2965 cm^−1^ and 1365 cm^−1^ were, respectively, due to anti-symmetric vibration and the characteristic peak of methyl in the hydroxypropyl group of HPβCD. The spectrum of the NG and HPβCD physical mixture (Figure 4A) was a superposition of their individual peaks that indicated almost all the characteristic peaks of both materials. In contrast, the NG/HPβCD inclusion complex (Figure 4A) showed a spectrum masking many characteristic peaks of NG that were clearly evident in their physical mixture profile. In the NG/HPβCD inclusion complex spectrum, the characteristic carbonyl peak of NG slightly shifted to lower wavenumbers, which could indicate the involvement of NG in forming new host–guest interaction with HPβCD. Moreover, the O-H vibrations of the inclusion complex shifted to lower wavenumbers concerning HPβCD while the O-H stretching mode of NG disappeared in this spectrum. This could be attributed to the involvement of OH groups of NG and HPβCD during the formation of the inclusion complex, which could result in alterations in the hydrogen bond network.

CDI-HPβNS (Figure 4B) displayed the characteristic peak of the carbonyl stretching vibrations at 1742 cm^−1^, which is attributed to the carbonyl group of the carbonate bond among CD monomers [33,37]. As expected, the spectrum of the physical mixture of NG and CDI-HPβNS (Figure 4B) was a superposition of their individual spectra. Characteristic peaks of NG and HPβNS did not show any significant shift in the physical mixture spectrum. On the contrary, the NG/CDI-HPβNS spectrum (Figure 4B) was similar to the CDI-HPβNS spectrum and many characteristic peaks of NG were masked by the nanosponge, although the C=O stretching of NG could be observed in NG/CDI-HPβNS spectrum with a 12 cm^−1^ increase in the wavenumber, which could indicate the strengthening of this bond as a result of nanosponge interaction with NG. In addition, the O-H stretching vibration of NG/CDI-HPβNS shifted to higher wavenumbers, which could be a result of the formation of hydrogen bonds through interacting NG with CDI-HPβNS.

#### 3.3.4. H NMR and ^13^C Cross-Polarization Magic Angle Spinning Solid-State NMR

**Figure 5:**
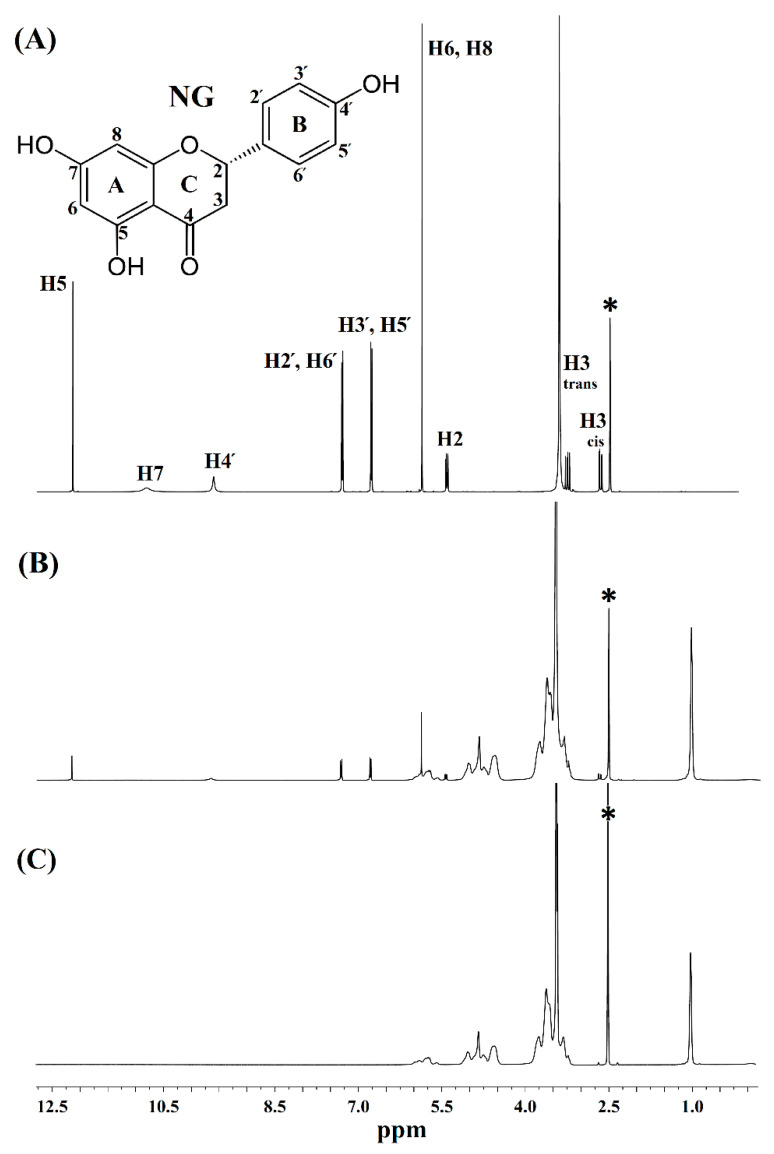
Proton nuclear magnetic resonance (^1^H NMR) spectra of (**A**) NG, (**B**) NG/HPβCD complex, and (**C**) HPβCD (* denotes DMSO-d_6_ peak at 2.5 ppm).

NMR spectroscopy has been widely used to study CD complexes since it is an effective tool for detecting the chemical shift changes during the host–guest interactions to confirm the complex formation [28]. In this study, the ^1^H NMR spectra of NG, HPβCD, and the NG/HPβCD complex (Figure 5) were recorded to investigate the proton chemical shifts that might be affected by the complex formation. Comparing the ^1^H NMR spectra of free NG (Figure 5A) and the NG/HPβCD complex (Figure 5B) demonstrated that all ^1^H protons in the NG chemical structure, including aromatic rings of A to C, displayed significant resonance alteration after the formation of the NG/HPβCD complex (Table 4). This could indicate that the entire NG molecule is involved in the formation of the NG/HPβCD complex. With the exception of H4′ and H7 related to the NG hydroxyl groups, other NG protons displayed an upfield shift, which could be due to a shielding effect of the valence electrons of HPβCD as a host molecule or an anisotropy effect after alteration of the molecular conformation due to the complex formation [39]. The hydroxyl group peak (H7) with low intensity in the NG spectrum, disappeared after complex formation, which can usually happen in the presence of residual water in ^1^H NMR. H4′, related to one of the NG hydroxyl groups, exhibited a significant downfield shift (Δδ = 0.023), which is a typical phenomenon for protons involved in hydrogen bonding. This indicates the role of hydrogen bonding in the formation of the NG/HPβCD complex. As previously indicated in this study, the Higuchi–Connors method showed a high stability constant for the formation of the NG/HPβCD complex. The Higuchi–Connors data. Along with the ^1^H NMR study, shows that NG has a high affinity for the formation of a stable host–guest complex with HPβCD through a process in which the hydrogen protons of the whole NG structure are involved and that this might be due to the insertion of the entire NG molecule into the cavity of HPβCD during the inclusion process. As reported in previous studies, the ^1^H NMR spectra of HPβCD is complicated by considerable overlap of the proton frequencies since, similar to most substituted CDs, HPβCD can be considered as a statistical mixture of different stereoisomers owing to its chemical modifications [43]. The ^1^H NMR spectrum of HPβCD (Figure 5C) contained signals that can be seen in NG/HPβCD spectrum with chemical shift alterations, but broad unresolved peaks of HPβCD, which does not allow the accurate following of changes in chemical shifts of its characteristic internal protons after the formation of host–guest complex; thus, their interpretation is not straightforward [44]. However, as mentioned earlier, alterations in the chemical shifts of NG protons after the host–guest complex formation in comparison with the chemical shift of the same protons in the free NG (Table 4) could reveal the inclusion of NG into the HPβCD cavity. Together, the results obtained from the ^1^H NMR spectra and the Higuchi–Connors method suggested that HPβCD formed a host–guest complex with NG through an inclusion phenomenon.

**Figure 6:**
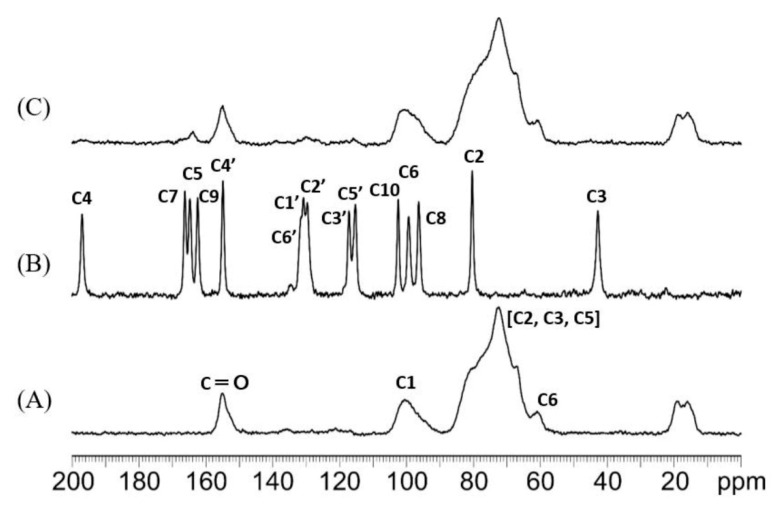
^13^C CPMAS NMR (0–200 ppm) spectra of CDI-HPβNS (**A**), NG (**B**), and NG/CDI-HPβNS (**C**), acquired with a spinning rate of 20 kHz at room temperature.

The ^13^C CPMAS solid-state NMR spectra of NG, CDI-HPβNS, and NG/CDI-HPβNS are presented in Figure 6. The spectrum of CDI-HPβNS (Figure 6A) exhibited several broad signals, including the characteristic peak of the carbonyl group at 155 ppm, which is related to carbonate cross-linkage among HPβCD molecules in the nanosponge structure. Castiglione et al., in a ^13^C CPMAS NMR study on CDI-based βCD nanosponges, observed four carbon resonances related to the glucose frame of βCD [45]. They reported chemical shifts of C1 at 102.8 ppm, C4 at 81.9 ppm, overlapping signals of C5, C3, and C2 at 73.2 ppm, and two broad peaks for C6 at 61.8 and 60.2 ppm [45]. CPMAS NMR of HPβNS in the present study (Figure 6A) showed the broad resonance almost at the same regions with chemical shift alterations less than 2 ppm, which could be assigned to the glucose frame of HPβCD. Therefore, it can be proposed that similar to βCD nanosponges with CDI as a crosslinker, the synthesis of HPβNS with the same crosslinker may not change the local environments of the HPβCD sugar ring after polymer formation [45].

**Figure 7:**
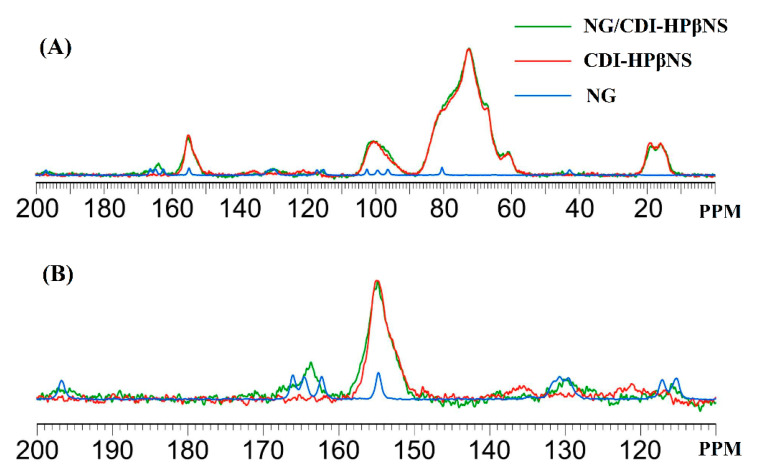
(**A**) Overlay of ^13^C CPMAS spectra of NG/CDI-HPβNS (in green), CDI-HPβNS (in red), and NG (in blue), in the 0–200 ppm range; (**B**) Detail in the 110–200 ppm range.

The NG spectrum (Figure 6B) indicated a set of well-resolved resonances for all carbons in its structure, which was in complete agreement with a previous report [46]. According to previous studies, the resonances of C4 = O (197 ppm), C8 (96.3 ppm), and C6 (99.2 ppm) in the NG spectrum typically appear in all flavonoids and the OH groups located at C4′ of ring B (154.8 ppm) and C7 of ring A (166.2 ppm) can form intermolecular hydrogen bonds while the C5-OH (164.7 ppm) mainly forms intramolecular hydrogen bonds [46]. All these peaks, in addition to other ^13^C CPMAS resonances of NG reported by Wawer et al., can be observed in the NG spectrum (Figure 6B). The spectrum of NG/CDI-HPβNS (Figure 6C) displays several broad signals at about 116 and 197 ppm, which, according to the well-resolved resonance of NG carbons, can be assigned to C5′ and the C4 carbonyl group of NG, respectively. Moreover, the NG/CDI-HPβNS spectrum showed several overlapping peaks at 130 ppm, which corresponded to the NG C2′, C1′, and C6′ resonances. Furthermore, several broad peaks were observed at 164 ppm in the NG/CDI-HPβNS spectrum, which might be related to the C9, C5, and C7 atoms of NG [46]. These resonances are not present in the spectrum of CDI-HPβNS and agree well with the typical resonances of NG. A comparison among the three spectra and the detail of the range 110–200 ppm is shown in Figure 7, in order to highlight the spectral features of the samples. Although the overlapping peaks and signals in the polymer spectrum cannot allow for following the exact chemical shift alterations of NG after loading in the nanosponge, the changes in the spectral features and the intensity of typical NG carbon resonances can indicate the interaction of NG with the CDI-HPβNS nanosponge, as well as the alterations in the NG chemical environment after the loading process.

#### 3.3.5. Particle Morphology by Scanning Electron Microscopy

**Figure 8:**
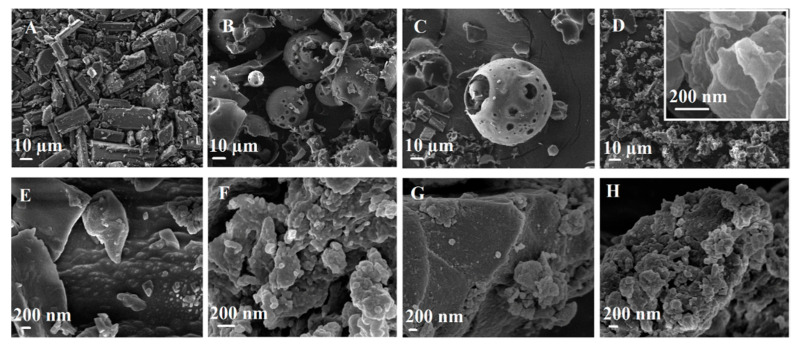
Scanning electron microphotographs of NG (**A**,**E**), HPβCD (**B**), NG + HPβCD physical mixture (**C**), NG/HPβCD complex (**D**), CDI-HPβNS (**F**), NG + CDI-HPβNS physical mixture (**G**) NG/CDI-HPβNS (**H**).

The scanning electron microscopy (SEM) was used to evaluate morphological changes in the pure NG and carriers as a result of complexation and NG loading. The SEM images of NG, carriers, their physical mixture, and their complex at the same weight ratio are shown in Figure 8.

Pure NG displayed a crystal shape with a cubic columnar form (Figure 8A) and a rough surface (Figure 8E). HPβCD (Figure 8B) was observed to have a spherical-like shape with different sizes, as well as some other irregular structures. HPβCD molecules formed spherical-like aggregates, as well as some other types in the solid state, as demonstrated in another report [44]. It should be mentioned that HPβCD, as a pure cyclodextrin molecule, does not have a porous structure, but when HPβCD molecules aggregate together they can form interesting spherical morphologies that seems porous and is detectable in SEM images at about 1 KX magnitude. This spherical morphology is not uniform in the whole sample since HPβCD aggregates are not uniform and this agrees with previous reports [44]. Both NG and HPβCD particle shapes remained intact in the SEM photograph of the physical mixture (Figure 8C). In contrast, considerable changes could be seen in the morphology of the NG/HPβCD complex (Figure 8D) without any similarity to the original shapes of the two pure materials from which it was formed. The NG/HPβCD complex showed an amorphous morphology with almost smooth surfaces that could evidence for the presence of a new complex i.e., NG/HPβCD. CDI-HPβNS (Figure 8F) presented porous irregular morphology and a rough surface. The intact morphology of both NG and CDI-HPβNS could be seen in the SEM image of their physical mixture (Figure 8G), but the crystal shape of NG could not be observed in the SEM photograph of NG/CDI-HPβNS (Figure 8H), which could confirm changes in the morphology of NG after loading and accommodating it in the porous structure of CDI-HPβNS.

### 3.4. In Vitro Release Study

As documented in the literature, the different metabolic profile of cancerous tumors causes excess protons to be released in the extracellular environment and reduce the pH from 7.40 to about 6.5–7 [47]. Furthermore, according to thermal medicine findings, a temperature increase in the tumor microenvironment of up to the fever range (mild hyperthermia) could improve the immunogenicity of tumor cells and enhance the efficacy of cancer treatment [48]. Therefore, it is valuable to compare the release pattern of nanocarriers in normal physiological conditions (37 °C in pH 7.4) and cancerous tissues conditions at fever temperature (39 °C in pH 6.6) to confirm the potential of nanocarriers for controlled release.

**Figure 9:**
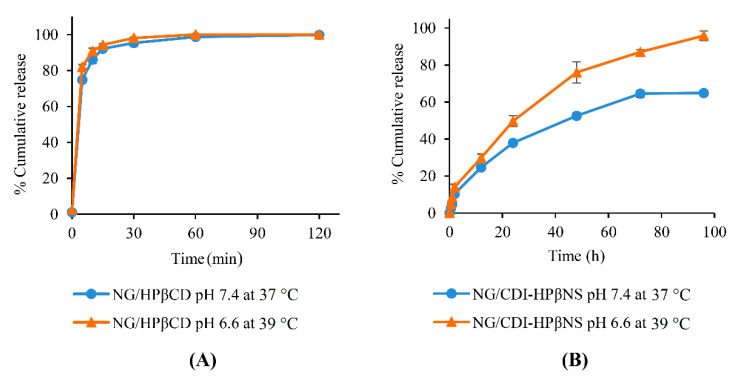
The release profile of NG/HPβCD (**A**) and NG/CDI-HPβNS (**B**) in two conditions: pH 7.4 at 37 °C and pH 6.6 at 39 °C.

As evident from Figure 9A, the NG/HPβCD complex displayed burst release in both conditions (37 °C in pH 7.4 and 39 °C in pH 6.6), with more than 95% of NG released in 30 min and the release process accomplished in 2 h. NG release from the NG/HPβCD complex in 39 °C and pH 6.6 was around 4% more than in normal conditions (*p* = 0.003), but there was no significant difference between both conditions between 1 and 2 h (*p* > 0.05). This burst pattern is in agreement with the previous literature, which indicated that drugs could be readily released from most CD complexes by simple dilution or competitive complexation [13,27].

In contrast, NG release from CDI-HPβNS in both conditions indicated a sustained release pattern without any initial burst effect (Figure 9B). This could suggest that there is no weakly adsorbed or uncomplexed NG on the surface of CDI-HPβNS. Moreover, the sustained pattern of the release process might be attributed to the porous matrix of CDI-HPβNS, which could be complexed strongly enough with NG to prevent the burst effect. NG release from CDI-HPβNS was significantly higher at all time points at pH 6.6 and 39 °C compared to the normal physiological pH and temperature (*p* < 0.05). Moreover, after 96 h NG/CDI-HPβNS released around 95% NG in pH 6.6 and 39 °C, which was approximately 31% more than the release percentage in normal physiological conditions (*p* < 0.05). This might be due to the lower pH and the higher temperature, which would make better conditions for higher solubilization and uncomplexation of NG in a way that the drug could be easier washed out by bathing fluid, even in the deep pores and capillaries of the CDI-HPβNS matrix [18,22]. Accordingly, CDI-HPβNS could be a good candidate for developing controlled and sustained-release drug delivery systems for cancer treatment.

### 3.5. Cytotoxicity Studies

**Figure 10:**
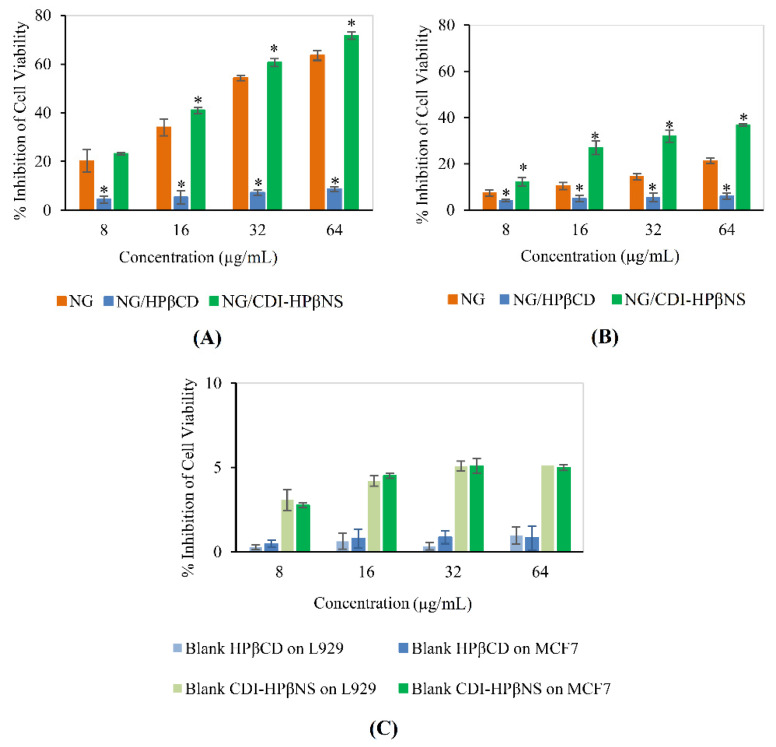
Cytotoxicity study of NG and NG-loaded carriers in L929 cells (**A**) and MCF7 cells (**B**), cytotoxicity study of blank carriers in L929 and MCF7 cells (**C**); * indicates significant difference (*p* < 0.05) between the NG/HPβCD or NG/CDI-HPβNS with NG via one-way ANOVA followed by Tukey post hoc test.

The biocompatibility and antiproliferative activity of NG, carriers, and NG-loaded carriers were evaluated through the MTT assay in MCF7 breast cancer cells (Figure 10A) and L929 murine fibroblasts (Figure 10B), respectively.

To prepare the NG positive control, NG was dissolved in DMSO since it is almost insoluble in aqueous medium and may aggregate. Then, this NG solution was dispersed in the cell medium (DMEM) so that the final DMSO concentration reached 0.1% (*v*/*v*). As established in the literature, DMSO at concentrations lower than 0.5% (*v*/*v*) have no toxic effects on the cells [5]. Free NG, as the positive control, led to a significant decrease in MCF7 cell viability in a concentration-dependent manner (*p* < 0.05). Similarly, in MCF7 cells, increasing the concentration of the NG/CDI-HPβNS nanosuspension significantly increased its cytotoxic effects (*p* < 0.05). NG/CDI-HPβNS resulted in a greater inhibition percentage at all concentrations compared to the NG positive control. In particular, at the concentrations of 16, 32, and 64 μg/mL, NG/CDI-HPβNS resulted in 41.04 ± 1.27, 60.61 ± 1.62, and 71.7 ± 1.51% inhibition, respectively, which was significantly more than NG positive control (*p* < 0.05). This could be due to the sustained release profile of CDI-HPβNS, which might increase the intracellular accumulation of NG in MCF7 cells and improve the anticancer efficacy.

In stark contrast, NG/HPβCD demonstrated toxicity at concentrations significantly lower than the NG positive control and NG/CDI-HPβNS at all concentrations (*p* < 0.05). The reason that could be mentioned for this case is that NG/HPβCD complex may decrease the NG uptake of MCF7 cells. This could be due to the fact that CDs could both enhance and restrict drug permeation through cellular membranes [13]. It seems that high HPβCD concentration decreased the thermodynamic potential of the NG and hampered its permeation through MCF7 cell membranes [13]. In contrast, it appears that NG/CDI-HPβNS particles could provide enough ligand-to-receptor interactions with MCF7 cells to enable internalization [49].

The highest concentration of NG/HPβCD in this experiment, containing 64 µg/mL NG, could inhibit the cell viability in the MCF7 as low as 8.67 ± 0.92%. Meanwhile, at this concentration, the NG/CDI-HPβNS demonstrated around 8.3-fold increase in the inhibition of MCF7 cell viability (71.7 ± 1.51%), indicating the remarkably higher anticancer potential of this nanosponge formulation towards MCF7 cell death. This improvement in the cytotoxic activity of drug-loaded nanosponges against cancer cells aligns with the previous studies. Dhakar et al. demonstrated a significant increase in the cytotoxicity of resveratrol and oxyresveratrol after using CDI nanosponges as a nanocarrier against DU-145 prostate cancer cells [33]. In another recent study, the results indicated that doxorubicin-loaded nanosponges decreased the half maximal inhibitory concentration (IC50) of MCF-7 cells up to 10 times compared to free doxorubicin and it improved the anti-cancer effectiveness in BALB-neuT mice [22].

In addition, NG, NG/HPβCD, and NG/CDI-HPβNS showed a similar inhibition pattern in the L929 cell line (Figure 10B), but with much lower toxicity. This could be attributed to the weak antiproliferative activity of citrus flavonoids e.g., NG against normal cell lines, while they were strongly active against cancerous tumors [50]. Since flavonoids have a dual function in ROS homeostasis, they could play as antioxidants in normal cells and potent pro-oxidants in cancer cells, resulting in activation of the apoptotic pathways [50].

Moreover, blank HPβCD and blank CDI-HPβNS did not show any significant toxicity, even at the highest concentration in L929 and MCF7 cells (Figure 10C). In addition, there was no significant difference between the effect of the blank carriers on L929 and MCF7 cell lines (*p* > 0.05). Earlier reports also demonstrated that blank CDI nanosponges did not exert any significant cytotoxic effect on SHSY-5Y human neuroblastoma cells and DU-145 prostate cancer cells [31,33]. These results could indicate the cytocompatibility of the blank carriers.

It is important to note that previous studies demonstrated both estrogenic and anti-estrogenic activity of NG in estrogen-deficient and -rich states of breast cancer cells respectively. These studies suggested that NG could act as a selective estrogen receptor modulator in the prevention and treatment of estrogen-related disorders [51,52]. This is especially crucial in menopausal women whose long-term administration of synthetic estrogen places them at risk for serious diseases such as endometrial and breast cancer [52]. Recently, another report indicated the considerable NG antitumor potential via the possible modulation of estrogen signaling, suggesting its therapeutic application in breast cancer patients [53]. Joining these results with the current study can suggest an important future perspective for the clinical application of NG/CDI-HPβNS as an oral or parenteral delivery system in preventing and treating estrogen receptor-positive (ER-positive) breast cancer. In the present work, NG/CDI-HPβNS exhibited an antiproliferative effect on an estrogen-dependent breast cancer cell line, namely MCF-7, which was significantly higher than free NG. Additionally, NG/CDI-HPβNS could overcome the limitations of the NG/HPβCD host–guest complex in the release pattern and antiproliferative activity. It also showed lower toxicity to normal L929 cells, but certainly further studies in normal human cell lines and in vivo efficacy experiments in appropriate animal models are still essential for confirming the translatability of these results to eventual clinical applications.

Moreover, it should be mentioned that NG, as a hydrophobic therapeutic agent, could be replaced with other hydrophobic drugs, including anticancer agents for loading in CDI-HPβNS. Tamoxifen and paclitaxel are two important examples of hydrophobic anticancer agents, which, in previous works, were loaded in β-cyclodextrin nanosponges and showed notable anticancer in vitro and in vivo results, as well as enhancement in pharmacokinetic parameters for breast cancer treatment [16,17,18]. In the current work, CDI-HPβNS was synthesized by hydroxypropyl β-cyclodextrin as the monomer is much more toxicologically benign compared to β-cyclodextrin. The results suggested that hydroxypropyl β-cyclodextrin CDI nanosponges with a porous structure and considerable loading capacity could act similar to β-cyclodextrin nanosponges in accommodating versatile hydrophobic agents, such as tamoxifen and paclitaxel, and could introduce these nanosponges as novel promising drug delivery systems.

## 4. Conclusions

In the present study, a host–guest complex of NG/HPβCD and three new HPβCD-based nanosponges were fabricated. Among the nanosponges, the NG/CDI-HPβNS with the highest NG loading capacity (19.42 ± 0.49%) was selected for comparison with the host–guest complex of NG/HPβCD concerning the loading capacity, particle size, morphology, stability, solubility, release profile, and antiproliferative effects. Through several characterization techniques, including TGA, DLS, ATR-FTIR, ^1^H NMR, ^13^C CPMAS Solid-State NMR, and SEM, the properties of blank and loaded carriers were evaluated and their formation was confirmed. The Higuchi–Connors method indicated an increase of more than 2300 times in the aqueous solubility of NG by HPβCD, as well as a high CE value and appropriate increase in dosage bulk, suggesting that the NG/HPβCD complex has good potential for use as a solid dosage form. Despite these benefits, the subsequent experiments showed that HPβCD did not improve the antiproliferative effect of NG through the host–guest complex. In contrast, when HPβCD plays the role of the building block to be crosslinked by CDI and form the nanosponge (CDI-HPβNS), its weaknesses were ameliorated from a pharmaceutical standpoint.

This could be considered a route from the aqueous soluble state (HPβCD) to a stable nanosuspension state (CDI-HPβNS) that brings several benefits, including enhancement of the loading capacity percentage (from 5.36 ± 0.32 (NG/HPβCD) to 19.42 ± 0.49 (NG/CDI-HPβNS)), sustained released pattern (in contrast to burst pattern of NG/HPβCD), and up to an 8.3-fold increase in antiproliferative effects against MCF-7 cancer cells. Moreover, both NG-loaded carriers had lower toxicity to L929 cells compared to MCF-7 cancer cells, while blank carriers did not show any significant toxicity on both cell lines. These findings suggested that CDI-HPβNS, as a result of forming a stable nanosuspension with considerable drug loading, sustained drug release profile, cytocompatible features, and the enhancement of NG antiproliferative effects, could be employed more efficiently in the role of a delivery vehicle with potential application in cancer treatment compared to the HPβCD host–guest complex.

Finally, it is noteworthy to mention what makes NG/CDI-HPβNS a superior nanomedicine to other choices. The facile process of nanocarrier synthesis, low cost of the HPβCD monomer, and no need for chemical modifications and catalysts can make scale-up stages uncomplicated and cost-effective. According to the results of this work, the sustained release profile of NG/CDI-HPβNS, accompanied by its higher toxicity to cancer cells compared to normal cells, can make it a promising choice for cancer treatment. The results also suggested that NG/CDI-HPβNS particles could be internalized by MCF7 cells much more effectively than the NG/HPβCD complex. Therefore, for the next step in our study, it will be worthwhile to focus on the cellular targeting and uptake capabilities of NG/CDI-HPβNS compared to NG/HPβCD via confocal microscopy and, finally, in vivo antitumor and pharmacokinetic studies on rats.

## Figures and Tables

**Table 1:** The synthesis conditions and materials for three types of HPβNS nanosponges.

Crosslinker	HPβCD (g)	Ratio *	Solvent (mL)	Catalyst	Conditions	Soxhlet Solvent	Ref.
PMDA	4.886	1:4	DMSO (20)	TEA	Magnet stirring at 25 ℃ for 3–4 h	Acetone	[29]
CDI	6.5	1:8	Anhydrous DMF (39)	-	Magnet stirring at 110 ℃ for about 12 h	Ethanol	[30]
Citric Acid	4	1:4	Deionized water (20)	SHP	in vacuum oven at 140 ℃ for 1 h then at 100 ℃ for 5–6 h	Acetone	[24]

* HPβCD:Crosslinker molar ratio.

**Table 2:** Percentage of NG loading capacity of 3 synthesized HPβNS in different solvents.

	NG Loading Capacity %		
HPβNS Type	Water	Water/Ethanol 50:50 *v*/*v*	Ethanol
PMDA-HPβNS	0.57 ± 0.27	2.17 ± 0.22	ND ^a^
CDI-HPβNS	1.76 ± 0.26	3.85 ± 0.43	19.42 ± 0.49
Citric Acid-HPβNS	0.41 ± 0.2	0.79 ± 0.13	4.64 ± 0.33

^a^ Not detectable.

**Table 3:** DLS and zeta potential data of NG/HPβCD and NG/CDI-HPβNS at the time of preparation (0 h) and after 7 days of storage (7d) at room temperature.

	NG/HPβCD (0 h)	NG/HPβCD (7d)	NG/CDI-HPβNS (0 h)	NG/CDI-HPβNS (7d)
Particle size (nm)	42.33 ± 11.16	84.03 ± 13.46	428.33 ± 1.99	453.13 ± 20.26
PDI	0.29 ± 0.02	0.29 ± 0.03	0.26 ± 0.01	0.27 ± 0.03
Zeta potential (mV)	1.91 ± 0.29	5.01 ± 0.37	−21.63 ± 2.14	−26.03 ± 3.42

**Table 4:** ^1^H NMR chemical shift difference (Δδ, ppm) of NG in the free state and in NG/HPβCD.

1H ^a^ Assignment	Chemical Shifts δ (ppm)		Δδ ^b^ (ppm)
	Free NG	NG/HPβCD Complex	
H2	5.444	5.439	−0.005
H3 (Cis)	2.688	2.682	−0.006
H3 (Trans)	3.228	3.221	−0.007
H5	12.153	12.146	−0.007
H6 or H8	5.879	5.867	−0.008
H7	10.827	-	-
H2′ or H6′	7.301	7.297	−0.004
H3′ or H5′	6.799	6.792	−0.007
H4′	9.623	9.646	0.023

^a^ DMSO-d_6_ signal was used as a reference at 2.500 ppm. ^b^ Δδ = δ NG/HPβCD complex—δ free NG.

## Data Availability

Not applicable.
